# Variations in policies for management of the third stage of labour and the immediate management of postpartum haemorrhage in Europe

**DOI:** 10.1111/j.1471-0528.2007.01377.x

**Published:** 2007-07-01

**Authors:** C Winter, A Macfarlane, C Deneux-Tharaux, W-H Zhang, S Alexander, P Brocklehurst, M-H Bouvier-Colle, W Prendiville, V Cararach, J van Roosmalen, I Berbik, M Klein, D Ayres-de-Campos, R Erkkola, LM Chiechi, J Langhoff-Roos, B Stray-Pedersen, C Troeger

**Affiliations:** a School of Nursing and Midwifery, University of Dundee Dundee, UK; b Department of Midwifery, City University London, UK; c INSERM UMR S149, Université PMC-Paris 6 Paris, France; d Perinatal Epidemiology Research Unit, Université Libre de Bruxelles Brussels, Belgium; e National Perinatal Epidemiology Unit, Oxford UK; f Department of Obstetrics and Gynaecology, Royal College of Surgeons of Ireland, Coombe Hospital Dublin, Ireland; g Hospital Clínic, IDIBAPS, University of Barcelona Barcelona, Spain; h Leiden University Medical Centre, Leiden the Netherlands; i Hungarian Society of Obstetrics and Gynaecology Budapest, Hungary; j Hanusch-Krankenhaus Gynakolog, University of Vienna Vienna, Austria; k Faculdade de Medicina da Universidade do Porto Porto, Portugal; l University Central Hospital of Turku Turku, Finland; m Unita di Obstetrica e gynecologia policlinica, University of Bari Bari, Italy; n Department of Obstetrics and Gynaecology, University of Copenhagen Copenhagen, Denmark; o Rikshospitalet, University of Oslo Oslo, Norway; p Pränatale Medizin, Universitäts Frauenklinik Basel, Switzerland

**Keywords:** Management policies, obstetric emergencies, postpartum haemorrhage, third stage of labour

## Abstract

**Background:**

The EUropean Project on obstetric Haemorrhage Reduction: Attitudes, Trial, and Early warning System (EUPHRATES) is a set of five linked projects, the first component of which was a survey of policies for management of the third stage of labour and immediate management of postpartum haemorrhage following vaginal birth in Europe.

**Objectives:**

The objectives were to ascertain and compare policies for management of the third stage of labour and immediate management of postpartum haemorrhage in maternity units in Europe following vaginal birth.

**Design:**

Survey of policies.

**Setting:**

The project was a European collaboration, with participants in 14 European countries.

**Sample:**

All maternity units in 12 countries and in selected regions of two countries in Europe.

**Methods:**

A postal questionnaire was sent to all or a defined sample of maternity units in each participating country.

**Main outcome measures:**

Stated policies for management of the third stage of labour and the immediate management of postpartum haemorrhage.

**Results:**

Policies of using uterotonics for the management of the third stage were widespread, but policies about agents, timing, clamping and cutting the umbilical cord and the use of controlled cord traction differed widely. For immediate management of postpartum haemorrhage, policies of massaging the uterus were widespread. Policies of catheterising the bladder, bimanual compression and in the choice of drugs administered were much more variable.

**Conclusions:**

Considerable variations were observed between and within countries in policies for management of the third stage of labour. Variations were observed, but to a lesser extent, in policies for the immediate management of postpartum haemorrhage after vaginal birth. In both cases, policies about the pharmacological agents to be used varied widely.

## Introduction

Despite the overall decline in maternal mortality in high-income countries, including those in Europe, since the middle of the 20th century, postpartum haemorrhage still makes a major contribution to maternal mortality and severe maternal morbidity in Europe.^1^–^3^

A collaborative project in the mid-1990s showed that there were wide differences between rates of severe pre-eclampsia, sepsis and postpartum haemorrhage in participating European countries, even though common definitions were used to collect the data.^1^ In this survey, overall differences between countries were dominated by differences in the incidence of postpartum haemorrhage. This ranged from 8.8 per 1000 deliveries in Finland to 0.7 in Austria. Possible explanations included differences in ascertainment,^1^ differences in the age distribution of women giving birth^3^ and differences in the ways in which care is provided and its quality.^4^

There is a prevailing view that the quality of care is particularly crucial where postpartum haemorrhage is concerned and that substandard care contributes to differences in the incidence of postpartum haemorrhage. Therefore, the adoption of appropriate policies, the availability of sufficient resources and the deployment of these resources to provide good care are central to the reduction of rates of severe postpartum haemorrhage.^4^ Despite this, little is known about relevant policies or practices in Europe. In response to these concerns, the five-part EUropean Project on obstetric Haemorrhage Reduction: Attitudes, Trial, and Early Warning System (EUPHRATES) was undertaken to address issues related to the prevention and management of postpartum haemorrhage.

The first component of this project was a survey undertaken to describe current obstetric and midwifery policies related to the prevention and management of postpartum haemorrhage in 14 countries of Europe and to inform the second part of the project, the development of a minimal European consensus for good practice related to the prevention and management of obstetric postpartum haemorrhage.^5^ The survey covered hospital policies in all the 14 countries and home birth policies in two of them. This article describes the methods used and the key findings about policies for the management of the third stage of labour and the immediate management of severe postpartum haemorrhage after vaginal birth in hospital and compares them with the available evidence.

## Methods

The project was a European collaboration, with participants from 12 European Union member states, Austria, Belgium, Denmark, Finland, France, Hungary, Ireland, Italy, the Netherlands, Portugal, Spain, and the UK plus Norway and Switzerland. Most countries sent a questionnaire to all their maternity units. The exceptions were Spain, where the survey covered maternity units in Catalonia; Portugal, where private maternity units were not surveyed; and France, where a purposive sample of six regions, reflecting a range of measures of maternal health and diversity of maternity care provision, was selected from regions willing to participate. Country coordinators were asked to provide available information about the units surveyed to enable the investigation of possible response bias in countries with low response rates.

A postal questionnaire was designed by the study steering group and refined and piloted by each participating country. It was sent out in 2003 and included questions about definition of postpartum haemorrhage, policies for management of the third stage of labour in vaginal birth and caesarean section, measurement of blood loss and management of postpartum haemorrhage. In each case, respondents were asked whether the policy in their unit was to use the specified interventions usually, occasionally, sometimes or never. In addition, questions were asked about the resources available in each maternity unit and about the level of activity in the preceding year, 2002.

The questionnaire was initially developed in English, and collaborators in each country decided whether translation into local languages would be beneficial. As a result, collaborators in France, Hungary, Norway, Portugal, Italy and Spain translated the questionnaire into their own languages. In Spain, where the survey was undertaken in Catalonia, the questionnaire was translated into both Catalan and Spanish languages, giving respondents a choice of language in which to reply. Because of the highly technical nature of the questions, formal back translation was not undertaken, but the questionnaires were checked against the English original.

Coordinators in each participating country established or obtained a list of maternity units to be surveyed. One questionnaire was sent to each unit, addressed to the midwife or obstetrician with overall management responsibility. Details are shown in Table 1. Two reminders were sent to nonrespondents, and additional prompts were given in some countries. The questionnaires were returned to coordinators within the country and then forwarded to City University, London, where data were entered onto an Access database and checks for inconsistency were undertaken. If any issues in the questionnaires needed clarification, the country coordinators were contacted for an explanation. The data were then analysed using the Statistical Package for the Social Sciences, version 12.0 for Windows (SPSS Inc., Chicago, IL, USA).

**Table 1:** Samples and response rates

Country	Maternity units sampled	Language	Number of units surveyed	Number of questionnaires received	Response rate (%)
Austria	All	English	104	33	31.7
Belgium	All	English	129	105	81.4
Denmark	All	English	29	23	79.3
Finland	All	English	33	33	100.0
France	Six regions	French	132	109	82.6
Hungary	All	Hungarian	98	98	100.0
Ireland	All	English	22	22	100.0
Italy	All	Italian	719	215	29.9
Netherlands	All	English	99	91	91.9
Norway	All	Norwegian	55	46	83.6
Portugal	All public maternity units	Portuguese	52	37	71.2
Spain	Catalonia	Catalan and Spanish	62	53	85.5
Switzerland	All	English	130	68	52.3
UK	All	English	354	242	68.4

This article describes the replies to five questions about the management of the third stage of labour and five questions about the immediate management of postpartum haemorrhage following vaginal birth.

The third stage of labour is defined as the period from birth of the baby until the placenta and its membranes are expelled. A sequence of procedures, known as ‘active management’, has been developed, but there is considerable variation in how these policies are defined and practised. As part of its consensus development process, the EUPHRATES group defined ‘active management’ as an intervention with the following three components:

1. Prophylactic administration of a uterotonic agent.
2. Clamping and cutting the cord immediately after birth or after it has stopped pulsating.
3. Controlled cord traction to aid the delivery of the placenta.

The analysis first looked separately at each component of active management and then at the extent to which responding units said that they used them all.

The questions about management of the third stage asked about policies for clamping and cutting the umbilical cord, draining the placenta, controlled cord traction and the prophylactic use of uterotonics, including their type and the timing of administration. In each case, the question asked whether the practice was used ‘usually’, ‘sometimes’, ‘rarely’ or ‘never’. If there was no reply to the question, this was interpreted as implying that there was no policy about using the practice.

Tables 2–4 are based on numbers of units in which the various interventions were said to be used ‘usually’, as this was assumed to imply either formally adopted or informally accepted policies. Where respondents replied ‘usually’ to two apparently mutually exclusive practices, this was tabulated separately.

**Table 2:** Policies for management of the third stage of labour after vaginal birth in maternity units from 14 European countries

	All units replying, *n*	Timing of cutting and clamping cord			Controlled cord traction, *n*(%)	Administration of prophylactic uterotonics, *n* (%)	Active management,** *n* (%)	Draining the placenta, *n* (%)
						
		Immediately after birth, *n* (%)	After the cord stops pulsating, *n* (%)	Other and not stated,* *n* (%)				
Austria	33	5 (15)	23 (70)	5 (15)	7 (21)	18 (55)	1 (3)	1 (3)
Belgium	105	92 (88)	11 (10)	2 (2)	45 (43)	93 (89)	36 (34)	34 (32)
Denmark	23	4 (17)	17 (74)	2 (9)	5 (22)	13 (57)	2 (9)	1 (4)
Finland	33	9 (27)	23 (70)	1 (3)	7 (21)	29 (88)	4 (12)	2 (6)
France	109	98 (90)	7 (6)	4 (4)	24 (22)	104 (95)	22 (20)	7 (6)
Hungary	98	20 (20)	66 (67)	12 (12)	12 (12)	89 (91)	5 (5)	3 (3)
Ireland	22	16 (73)	5 (23)	1 (5)	21 (95)	22 (100)	17 (77)	3 (14)
Italy	215	142 (66)	43 (20)	30 (14)	28 (13)	197 (92)	20 (9)	14 (7)
Netherlands	91	67 (74)	21 (23)	3 (3)	41 (45)	86 (95)	33 (36)	0 (0)
Norway	46	11 (24)	30 (65)	5 (11)	18 (39)	33 (72)	5 (11)	7 (15)
Portugal	37	33 (89)	1 (3)	3 (8)	19 (51)	31 (84)	13 (35)	9 (24)
Spain	53	40 (75)	7 (13)	6 (11)	13 (25)	45 (85)	7 (13)	8 (15)
Switzerland	68	47 (69)	10 (15)	11 (16)	31 (46)	60 (88)	25 (37)	2 (3)
UK	242	186 (77)	31 (13)	25 (10)	210 (87)	232 (96)	182 (75)	8 (3)

* A few units had more than one ‘usual’ policy or had a policy of cutting the cord ‘at another time’.

** Usually cut the cord immediately after birth or after the cord stops pulsating, perform ‘controlled cord traction’ and administer prophylactic uterotonics.

**Table 4:** Policies about use of mechanical methods for controlling postpartum haemorrhage in maternity units from 14 European countries

Country	All units replying, *n*	Massage the uterus, *n* (%)	Catheterise the bladder, *n* %)	Bimanual compression of uterus, *n* (%)
Austria	33	29 (88)	27 (82)	16 (48)
Belgium	105	100 (95)	79 (75)	44 (42)
Denmark	23	22 (96)	9 (39)	13 (57)
Finland	33	30 (91)	9 (27)	14 (42)
France	109	98 (90)	87 (80)	40 (37)
Hungary	98	82 (84)	82 (84)	35 (36)
Ireland	22	22 (100)	21 (95)	8 (36)
Italy	215	201 (93)	118 (55)	45 (21)
Netherlands	91	75 (82)	84 (92)	14 (15)
Norway	46	42 (91)	29 (63)	21 (46)
Portugal	37	37 (100)	26 (70)	25 (68)
Spain	53	52 (98)	36 (68)	21 (40)
Switzerland	68	58 (85)	49 (72)	31 (46)
UK	242	229 (95)	229 (95)	82 (34)

The questions about the immediate management also asked whether the practices were used ‘usually’, ‘sometimes’, ‘rarely’ or ‘never’ and were analysed in the same way. The questions about use of pharmacological or other agents for the immediate management of haemorrhage asked respondents to name the agent which would be used first and which would be used next if bleeding continued.

**Figure 1:**
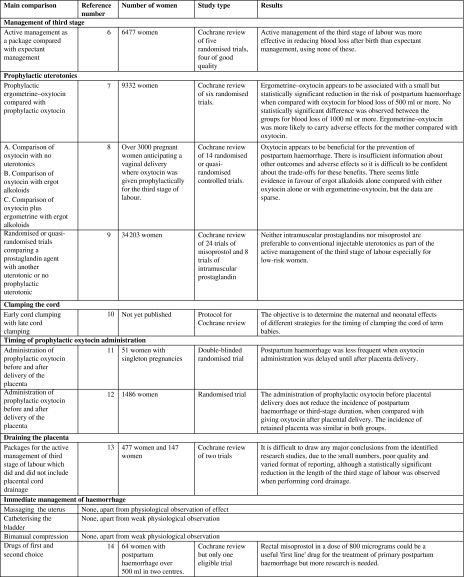
Summary of Cochrane reviews and other evidence.

The responses were compared using both asymptotic and Monte Carlo estimates of Pearson’s chi-square, calculated using StatXact.7 (Cytel Inc., Cambridge, MA, USA). The results were compared with the evidence available from Cochrane reviews, summarised in Figure 1.

## Role of the funding source

The project was funded by the European Union under Framework 5. The sponsor of the study had no role in study design, data collection, data analysis, data interpretation or the writing of the report. The steering group had full access to all the data in the study and had final responsibility for submission for publication on behalf of the EUPHRATES Group.

## Results

### Response rates and investigation of response bias

The number of questionnaires sent out ranged from 22 in Ireland to 719 in Italy. This wide range resulted from differences in the overall numbers of births and in the sizes of maternity units in the participating countries. Response rates varied from 29.9% in Italy and 31.7% in Austria to 100% in Hungary, Finland and Ireland, with response rates above 65% in 11 out of 14 countries, as Table 1 shows, with only Austria, Italy and Switzerland having rates below this level. In Italy, response rates in public and private hospitals were similar. Among public hospitals, for which fuller information was available, there was considerable regional variation, with rates around 50% in the regions of Lombardia, Veneto and Friuli-Venezia Giulia. Elsewhere, response rates ranged from zero among the nine maternity units in Umbria to around 40% in the regions of Puglia and Basilicata. In Switzerland, response rates were higher from the public sector than from the private sector, with only a few private hospitals replying. No information was available to investigate possible response bias in Austria.

In the statistical tests undertaken on the data in Tables 1–5, *P* values were below 0.0005 in each case, demonstrating wide differences in policies between the participating countries.

**Table 5:** Drug of first choice for management of postpartum haemorrhage in maternity units from 14 European countries

Country	All units replying, *n*	Oxytocin, *n* (%)	Syntometrine, *n* (%)	Ergometrine, *n* (%)	Injectable prostaglandins, *n*(%)	Misoprostol, *n* (%)	Other and not stated, *n* (%)
Austria	33	23 (70)	1 (3)	3 (9)	6 (18)	0 (0)	0 (0)
Belgium	105	56 (53)	0 (0)	29 (28)	18 (18)	0 (0)	0 (0)
Denmark	23	17 (74)	0 (0)	1 (4)	0 (0)	5 (22)	0 (0)
Finland	33	24 (73)	1 (3)	1 (3)	1 (3)	6 (18)	0 (0)
France	109	87 (80)	0 (0)	1 (1)	4 (4)	16 (15)	1 (1)
Hungary	98	80 (82)	0 (0)	6 (6)	11 (11)	1 (1)	0 (0)
Ireland	22	10 (45)	9 (41)	2 (9)	0 (0)	1 (5)	0 (0)
Italy	215	172 (80)	0 (0)	21 (10)	10 (5)	9 (4)	2 (1)
Netherlands	91	51 (56)	0 (0)	19 (21)	19 (21)	2 (2)	0 (0)
Norway	46	39 (85)	0 (0)	2 (4)	1 (2)	4 (9)	0 (0)
Portugal	37	35 (95)	0 (0)	1 (3)	0 (0)	1 (3)	0 (0)
Spain	53	29 (55)	0 (0)	23 (43)	1 (2)	0 (0)	0 (0)
Switzerland	68	41 (60)	1 (1)	13 (19)	4 (6)	8 (12)	1 (1)
UK	242	68 (28)	44 (18)	102 (42)	16 (7)	3 (1)	9 (4)

### Managing the third stage of labour

#### Cutting and clamping the cord and draining the placenta

Maternity units were asked about the point at which the cord was clamped and cut. Table 2 shows that between 66 and 90% of units in Belgium, France, Ireland, Italy, the Netherlands, Portugal, Spain, Switzerland and the UK had policies of clamping and cutting the cord immediately after the birth, but between 65 and 74% of units in Austria, Denmark, Finland, Hungary and Norway had policies of waiting until the cord stopped pulsating. More than 10% of units in Austria, Hungary, Italy and Switzerland did not give a stated policy. Five units, three in the UK, one in Spain and one in Norway, said they usually cut the cord at both times and were included in those with no stated policies.

Replies to the question about draining the placenta, which is not seen as part of ‘active management’, suggested that this practice was not widespread in any country, except in Belgium, where it was policy in 32% of maternity units, and Portugal, where it was a policy in 24%. Apart from this, it was policy in approximately 14% of units in Ireland, 15% of units in Norway and Spain and between 0 and 7% in the other countries, as Table 2 shows.

#### Controlled cord traction

Policies of controlled cord traction were also common, but their extent differed markedly between countries. Controlled cord traction was policy in 87% of units in the UK, in 95% of those in Ireland and from 39 to 51% of units in Belgium, the Netherlands, Norway, Portugal and Switzerland. In the other participating countries, it was said to be policy in between 12 and 25% of units.

#### Prophylactic use of uterotonics

It was policy to use uterotonics prophylactically in between 72 and 100% of units in most participating countries apart from Austria and Denmark where 55 and 57% of the units, respectively, reported a policy of using them, as Table 2 shows.

Policies for the timing of prophylactic administration of uterotonics diverged considerably, as Table 3 shows. In the 68% of units in the UK and Ireland, it was policy to administer them immediately after the delivery of the anterior shoulder, while between 62 and 87% of units in Denmark, Finland, the Netherlands, Norway and Switzerland favoured administering uterotonics immediately after birth. Between 69 and 77% of units in Italy, Portugal and Spain had policies of administering them after the delivery of the placenta. In Belgium, France and Hungary, policies differed both between and within units. In particular, 10% of units in Belgium and 19% in France cited more than one policy. It is possible that, in these units, two doses of uterotonic are administered at different stages, with the first dose being administered either at the delivery of the anterior shoulder or immediately after the birth and the second after the expulsion of the placenta.

**Table 3:** Policies on type and timing of administration of prophylactic uterotonics in vaginal birth in maternity units from 14 European countries

Country	All units using prophylactic uterotonics, *n*	Type of prophylactic uterotonic*					Timing of prophylactic uterotonic administration**				
			
		Oxytocin alone, *n* (%)	Syntometrine, *n* (%)	Ergometrine alone, *n* (%)	More than one ‘usual’, *n* (%)	Not stated, *n* (%)	After delivery of the anterior shoulder, *n* (%)	Immediately after birth, *n* (%)	After delivery of placenta, *n* (%)	More than one ‘usual’ time given, *n* (%)	No time given, *n* (%)
Austria	18	10 (56)	1 (6)	0 (0)	4 (22)	3 (17)	4 (22)	4 (22)	0 (0)	1 (6)	9 (50)
Belgium	93	49 (53)	0 (0)	24 (26)	13 (14)	7 (8)	23 (25)	26 (28)	28 (30)	9 (10)	5 (5)
Denmark	13	10 (77)	1 (8)	0 (0)	1 (8)	1 (8)	1 (8)	8 (62)	3 (23)	0 (0)	1 (8)
Finland	29	18 (62)	0 (0)	4 (14)	7 (24)	0 (0)	0 (0)	24 (83)	1 (3)	1 (3)	1 (3)
France	104	96 (92)	0 (0)	0 (0)	1 (1)	7 (7)	32 (31)	9 (9)	31 (30)	20 (19)	11 (11)
Hungary	89	64 (72)	0 (0)	0 (0)	11 (12)	14 (16)	2 (2)	45 (51)	17 (19)	4 (4)	21 (24)
Ireland	22	4 (18)	14 (64)	0 (0)	2 (9)	1 (5)	15 (68)	6 (27)	0 (0)	0 (0)	1 (5)
Italy	197	72 (37)	7 (4)***	34 (17)	68 (35)	16 (8)	6 (3)	22 (11)	150 (76)	5 (3)	14 (7)
Netherlands	86	78 (91)	0 (0)	1 (1)	4 (5)	3 (3)	3 (3)	75 (87)	3 (3)	2 (2)	3 (3)
Norway	33	30 (91)	0 (0)	0 (0)	0 (0)	2 (6)	0 (0)	23 (70)	6 (18)	0 (0)	3 (9)
Portugal	31	29 (94)	0 (0)	0 (0)	1 (3)	1 (3)	1 (3)	0 (0)	24 (77)	1 (3)	5 (16)
Spain	45	41 (91)	0 (0)	0 (0)	1 (2)	3 (7)	4 (9)	3 (7)	31 (69)	4 (9)	3 (7)
Switzerland	60	56 (93)	0 (0)	0 (0)	2 (3)	2 (3)	7 (12)	49 (82)	1 (2)	2 (3)	1 (2)
UK	232	12 (5)	201 (87)	1 (0)	8 (3)	9 (4)	158 (68)	59 (25)	2 (1)	2 (1)	11 (5)

* Total is not always 100% because in a few units, an ‘other type’ of prophylactic uterotonic was used.

** Total is not always 100% because in a few units, prophylactic uterotonics were administered ‘at another time’.

*** Syntometrine is not available in Italy. One unit had used it experimentally. The others had used combinations of oxytocin and ergometrine separately.

In units with a policy of administering prophylactic uterotonics, the agent most commonly used was oxytocin alone. This was used in more than 90% of units in France, the Netherlands, Norway, Portugal, Spain and Switzerland. The exception was in the UK and Ireland, where Syntometrine®, a fixed combination of oxytocin and ergometrine, was used in 87 and 64% of units, respectively. This formulation is not licensed for use in any other participating countries, possibly apart from Austria. Of the seven units in Italy reporting using it, one had used it experimentally and the others had used combinations of oxytocin and ergometrine separately. This also could have been the case in the units in Denmark and Austria, which reported using it prophylactically.

Much smaller proportions of units, ranging from 14 to 26%, in Belgium, Finland and Italy had policies of using only ergometrine. In addition, in these countries plus Hungary and Austria, between 12 and 35% of units said they used more than one type of prophylactic uterotonic, the usual combination being oxytocin and ergometrine. In France, the coordinator reported that some of these replies arise from policies of using ergometrine after the placenta is delivered, having used oxytocin at an earlier stage.

#### Active management

The proportions of units with policies of using the full package of active management of the third stage of labour described earlier were much lower. While 77% of units in Ireland and 75% of those in the UK had a set of policies which fitted the definition of active management, only 34–37% of units in Belgium, the Netherlands, Portugal and Switzerland and fewer than 20% of those in the other participating countries had policies of active management, as Table 2 shows. It this case, units which answered ‘usually’ to both timings for cutting and clamping the cord were included.

### Procedures for immediate management of postpartum haemorrhage

From 82 to 100% of units in every country had a policy of massaging the uterus on occasions when postpartum haemorrhage occurs, as Table 4 shows. The proportion of units in each country which had a policy of catheterising the bladder was 80% or more in Austria, France, Hungary, Ireland, the Netherlands and the UK. It ranged from 55 to 75% in Belgium, Italy, Norway, Portugal, Spain and Switzerland. In contrast, only 39% of units in Denmark and 27% in Finland reported this policy. Between 34 and 68% of units in all countries had policies of using bimanual compression of the uterus. The exceptions were in the Netherlands and Italy, where only 15 and 21% of maternity units, respectively, reported this policy.

#### Administration of pharmacological agents

There were considerable differences both between and within countries in their choice of drugs for the immediate management of postpartum haemorrhage. The proportion of all units using oxytocin alone as their first choice ranged from 28% in the UK to 95% in Portugal. This included 80–95% of the units in France, Hungary, Italy, Norway and Portugal, as Table 5 shows. Syntometrine® was used as a drug of first choice in 18% of units in the UK and 41% of those in Ireland but was used in only one unit in each of Austria, Finland and Switzerland. As Syntometrine® is not licensed for use in these countries, possibly except in Austria, it could be that these units had a policy of using oxytocin and ergometrine in combination. Slightly more than 40% of the units in UK and Spain reported using ergometrine alone as first line management. About one-fifth of units in Austria, Belgium and the Netherlands had policies of using injectable prostaglandins, while 22% of units in Denmark, 18% of those in Finland and 15% in France had a policy of using misoprostol.

There was similar diversity in the drug of second choice, should the postpartum haemorrhage not be controlled with the first. In Belgium, France, Hungary, Italy, the Netherlands and Portugal, prostaglandins were the agent of second choice in 50–80% of units. Ergometrine alone was the second choice in more than two-fifths of units in Austria and Spain. Only units in the UK and Ireland gave oxytocin alone as their second choice. This was the case for 46% of units in the UK and 32% of those in Ireland. Only 306 units, just more than one-quarter, specified a third choice of drug, and 259 of these specified either injectable prostaglandins or misoprostol.

## Discussion

This survey showed considerable differences both between countries and between maternity units within European countries in their stated policies for management of the third stage of labour and the immediate management of postpartum haemorrhage. Countries were used as sampling units, both for administrative reasons and also because it was thought that national professional organisations would be likely to issue guidelines and policy recommendations to their members. In fact, only Hungary, the Netherlands, Norway, Spain and Scotland had guidelines on the subject at the time the survey was undertaken, although guidelines have been published subsequently in Denmark and France. The status and influence of these guidelines and the professional and other organisations issuing them is known to vary between countries. For these reasons, one of the components of this project was the development of a consensus statement.^5^

Because the study included countries where collaborators could be readily identified, it covered 11 of the member states of the European Union at the time it was undertaken; Hungary, which has subsequently joined, plus two nonmembers, Switzerland and Norway. In most countries, all maternity units were surveyed. The exceptions were France, Portugal and Spain. In Portugal, private hospitals were not surveyed because they usually only rent out facilities for deliveries and have no hospital policies on clinical issues. These represented about 5.5% of births in the country at the time the survey was carried out. In France, six regions, containing about 20% of units in France, were surveyed. They were purposively selected to be representative of different parts of the country. The units in these regions were compared to all units in France using data from the 2003 National Perinatal Survey and found to be representative in terms of unit size and level of care and status as university, other public or private units. In Spain, the coordinator chose, for administrative convenience, to survey all units in Catalonia. Two countries had exceptionally low response rates, Italy where, because of illness on the part of the coordinator, reminders were not sent out, and Austria.

It has been shown that many maternity units in most countries do not use the full package of active management but do use some of its components. Most have policies about cutting and clamping the cord, either immediately after the birth or as soon as the cord stops pulsating, but differences in policies about controlled cord traction are much wider. Policies of using uterotonics are very widespread, and differences again relate to the timing of administration together with the pharmacological agent used.

At first sight, the information in Figure 1 suggests that Cochrane reviews provide clear evidence about the active management of the third stage of labour, but closer examination raises a number of questions. Much of the debate and research about the management of the third stage of labour is based on an assumed dichotomy between ‘active’ and ‘expectant’ management. Definitions of active management usually imply a combination of the use of uterotonics, early cord clamping and active efforts to deliver the placenta following delivery, but the definition is neither unambiguous nor agreed in practice.^9^

A particularly striking difference was in policies about use of uterotonics. While in the UK and the Irish Republic most units had a policy of using Syntometrine®, it appears that it was not available in any of the other participating countries. There may be a number of reasons for this. For example, Belgium and Spain have policies of not licensing products which contain two compounds. As oxytocin and ergometrine are both available, then it could be argued that Syntometrine® is unnecessary. In addition, the relevant Cochrane review clearly states that there are more unpleasant adverse effects with Syntometrine® than with oxytocin.^7^ So this might make some countries reluctant to license Syntometrine®.

Although there have been a considerable number of randomised trials of care in the third stage of labour, most have either compared the various pharmacological agents used or compared expectant with active management as a whole.^6^–^9^ Very few studies have attempted to examine the relative contribution of the individual components of active management. The policy of draining the placenta is not widespread but has been the subject of a Cochrane review, which identified only four trials, two of which were excluded on the grounds of inadequate reporting.^13^ Thus, there are still major gaps in the evidence about the optimal management of the third stage.

The survey showed similarities in policies for the immediate management of postpartum haemorrhage but considerable differences in the choice of pharmacological agents. Variation in policies for the management of postpartum haemorrhage was also found in a survey of maternity units conducted in 2000–01 in the UK.^15^ Evidence about management of postpartum haemorrhage is extremely sparse. No research has been carried out to establish the usefulness or efficacy of mechanical methods for the management of postpartum haemorrhage caused by uterine atony, let alone about postpartum haemorrhage resulting from tears or ruptures. Bimanual compression of the uterus tends to be used in environments, such as a home birth, where there is no medical support. It maintains homeostasis until the arrival of medical help.^16^,^17^ A Cochrane review of drugs for immediate management of postpartum haemorrhage identified just one small trial which suggested that rectal misoprostol could be a useful first line treatment but that further trials were needed.^14^

Since this survey was undertaken, a number of trials of use of misoprostol, both for the third stage of labour and for the immediate management of haemorrhage, have been published, so this might have led to changes in policies. On the other hand, a review by the International Confederation of Midwives and the International Federation of Gynaecology and Obstetrics has suggested that as far as the management of the third stage is concerned, the main advantages of misoprostol are in resource-poor countries where it is difficult to maintain oxytocin at the required temperature.^18^

Despite these limitations, this survey has provided new information about differences in policies for management of the third stage of labour and the immediate management of postpartum haemorrhage in Europe.

This project did not attempt to fill the gap in information about the relationship between units’ stated policies and their actual practice. A comparison of actual practice in units with the same stated policy was undertaken in 1999 in an international survey. This observed practice in the management of the third stage of labour in approximately 30 successive vaginal deliveries in 15 university-based obstetric centres with policies of active management of the third stage in two high-income and 13 low-income countries. It found considerable variations within and between units in the use of active management.^19^ This suggests that if there were guidelines for management, they were not clearly implemented, although compared with Europe, other issues may be involved, notably the availability of oxytocic drugs. Nevertheless, the existence of guidelines can affect practice, especially if practice is audited.^20^,^21^

## Conclusions

Considerable differences in policies for managing the third stage of labour were observed both between and within countries. For policies which were widespread, notably cutting and clamping the cord and use of uterotonics, there were differences in timing and use of pharmacological agents. There were greater similarities in policies for the immediate management of postpartum haemorrhage, but considerable differences between and within countries in the choice of pharmacological agents. Further research is needed to ascertain whether policies are being translated into practice and assess their associations with the incidence and consequences of postpartum haemorrhage.

## Contribution to authorship

S.A. initiated the collaborative project. C.W., A.M., C.D-T., W-H.Z., S.A., P.B., M.-H.B.C., W.P. and V.C. participated in the design of the survey and the questionnaire. J.vR., I.B., M.K., D.A-de-C., R.E., L.M.C., B.S-P., and C.T. commented on the design of the survey and the questionnaire. C.W., A.M., C.D-T., W-H.Z., S.A., M.-H.B.C., W.P., V.C., J.vR., I.B., M.K., D.A-de-C., R.E., L.M.C., B.S-P. and C.T. were involved in undertaking the survey in their countries and J.L-R. participated in the survey. C.W. and A.M. cleaned and analysed the data and drafted the paper. All the authors took part in the revision of the paper and have seen and approved the final version. None have any conflicts of interest.

## EUPHRATES steering group

S.A. (Obstetrician, Epidemiologist), W-H.Z. (Epidemiologist), M.-H.B-C. (Epidemiologist), G.B. (Epidemiologist), C.D-T. (Epidemiologist), A.M. (Epidemiologist, Statistician), W.P. (Obstetrician), V.C. (Obstetrician), P.B. (Obstetrician, Epidemiologist) and C.W. (Midwife).
